# Coherent microwave-photon-mediated coupling between a semiconductor and a superconducting qubit

**DOI:** 10.1038/s41467-019-10798-6

**Published:** 2019-07-08

**Authors:** P. Scarlino, D. J. van Woerkom, U. C. Mendes, J. V. Koski, A. J. Landig, C. K. Andersen, S. Gasparinetti, C. Reichl, W. Wegscheider, K. Ensslin, T. Ihn, A. Blais, A. Wallraff

**Affiliations:** 1 Department of Physics, ETH Zürich, CH-8093 Zürich, Switzerland; 2 Institut quantique and Department de Physique, Université de Sherbrooke, Sherbrooke, Québec J1K 2R1 Canada; 3 Canadian Institute for Advanced Research, Toronto, ON Canada; 4 Present Address: Instituto de Física, Universidade Federal de Goiás, Goiânia, Go CEP 74.690-900 Brazil

**Keywords:** Quantum information, Superconducting devices, Qubits

## Abstract

Semiconductor qubits rely on the control of charge and spin degrees of freedom of electrons or holes confined in quantum dots. They constitute a promising approach to quantum information processing, complementary to superconducting qubits. Here, we demonstrate coherent coupling between a superconducting transmon qubit and a semiconductor double quantum dot (DQD) charge qubit mediated by virtual microwave photon excitations in a tunable high-impedance SQUID array resonator acting as a quantum bus. The transmon-charge qubit coherent coupling rate (~21 MHz) exceeds the linewidth of both the transmon (~0.8 MHz) and the DQD charge qubit (~2.7 MHz). By tuning the qubits into resonance for a controlled amount of time, we observe coherent oscillations between the constituents of this hybrid quantum system. These results enable a new class of experiments exploring the use of two-qubit interactions mediated by microwave photons to create entangled states between semiconductor and superconducting qubits.

## Abstract (web summary)

Hybrid quantum devices combine different platforms with the prospect of exploiting the advantages of each. Scarlino et al. demonstrate strong, coherent coupling between a semiconductor qubit and a superconducting qubit by using a high-impedance superconducting resonator as a quantum bus.

## Introduction

Single electron spins confined in semiconductor quantum dots (QDs) can preserve their coherence for hundreds of microseconds in ^28^Si^1,2^, and have typical relaxation times of seconds^3,4^. This property can be explored, for example, to build memories for quantum information processors in hybrid architectures combining superconducting qubits and spin qubits. Typically, semiconductor qubit–qubit coupling is short range, effectively limiting the interqubit distance to the spatial extent of the wavefunction of the confined particle, which is a significant constraint toward scaling to reach dense 1D or 2D arrays of QD qubits. Strategies to interconnect semiconductor qubits include the control of short-range interactions through the direct overlap of electronic wavefunctions^5–7^, the direct capacitive coupling between QDs^8^, enhanced by floating metallic gates^9^, shuttling of electrons between distant QDs by surface acoustic waves^10,11^, by time-varying gate voltages^12^ and by fermionic cavities^13^. An alternative approach which allows for long-range qubit–qubit interaction, inspired by superconducting circuit quantum electro-dynamics (QED)^14^, and recently explored also for semiconductor QDs^15–17^, is to use microwave photons confined in superconducting resonators to mediate coupling between distant qubits. In this approach, the microwave resonator not only acts as a quantum bus, but also allows for quantum nondemolition qubit readout^18–20^.

With the well established strong coupling of superconducting qubits to microwave resonators^14^ and the recently achieved strong coupling to charge states in semiconductor double dot structures^21,22^, it is now possible to create a microwave photon-based interface between superconducting and semiconducting qubits mediated by a joint coupling resonator. A similar strategy has been explored in hybrid structures interfacing a transmon qubit with excitations of a spin-ensemble of NV centers in diamonds^23–25^ and of collective spins (magnons) in ferromagnets^26–28^. Furthermore, direct coupling between a superconducting flux qubit and an electron spin ensemble in diamond was investigated^29^. In these works the strong coupling regime was achieved with ensembles, for which the coupling strength scales with the square root of the number of two-level systems interacting with the resonator mode.

Here, we explore the coupling of the charge degree of freedom of a single electron confined in a double QD (DQD) to a superconducting transmon qubit in the circuit QED architecture^14^. The coherent coupling between dissimilar qubits over a distance of a few hundred micrometers is mediated by virtual microwave photon excitations in a high impedance SQUID array resonator, which acts as a quantum bus. We demonstrate resonant and dispersive interaction between the two qubits mediated by real and virtual photons, respectively. We extract a coupling strength of ~36 MHz (~128 MHz) between the bus resonator and the DQD (transmon) around the frequency of ~3.7 GHz. With a frequency detuning of ~370 MHz from the resonant frequency of the bus resonator, we spectroscopically observe a qubit avoided crossing of about ~21 MHz. The strength of the virtual-photon mediated interaction is extracted from measurements of coherent qubit population oscillations. The methods and techniques presented here have the potential to be transferred to QD devices based on a range of material systems and can be beneficial for spin-based hybrid systems.

## Results

### Sample design and basic circuit characterization

**Fig. 1:**
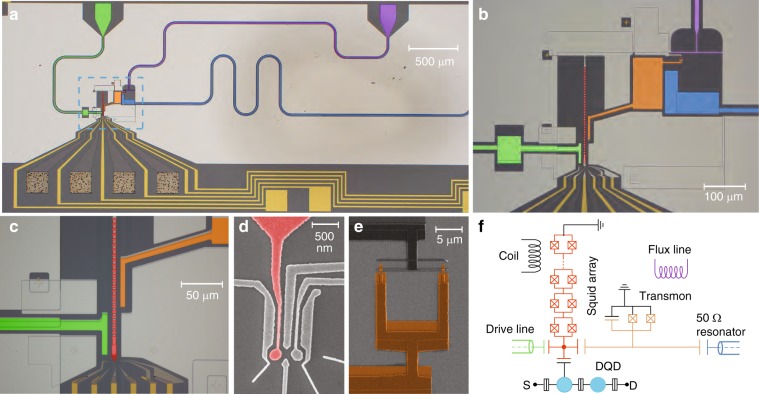
Sample and simplified circuit diagram. **a** False color optical micrograph of the device showing the substrate (dark gray), the Al superconducting structures forming the groud plane (light gray), the DQD Au gate leads (yellow), the SQUID array resonator (red), its microwave feedline (green), the single island transmon (orange), its readout 50 Ω coplanar waveguide resonator (blue), and the flux line (purple). **b** Enlarged view of the sample area enclosed by the blue dashed line in panel (**a**). **c** Enlarged view of the coupling side of the SQUID array. **d** Electron micrograph of the DQD showing its electrostatic top gates (Al-light gray) and the plunger gate coupled to the SQUID array (red). **e** Electron micrograph of the transmon SQUID. **f** Circuit diagram schematically displaying the DQD [with its source (*S*) and drain (*D*) contact], capacitively coupled to the SQUID array resonator, which in turn is coupled to the transmon. The transmon and the SQUID array are respectively capacitively coupled to a 50 Ω CPW resonator and microwave feedline. Their resonance frequencies can be tuned by using a flux line and a coil schematically shown in the circuit diagram. The color code is consistent with the optical micrographs

To perform our experiments, we integrate four different quantum systems into a single device: a semiconductor DQD charge qubit, a superconducting qubit, and two superconducting resonators (see Fig. 1a). One resonator acts as a quantum bus between the superconducting and the semiconductor qubits and the other one as a readout resonator for the superconducting qubit. In this way, the functionality for qubit readout and coupling is implemented using two independent resonators at different frequencies, allowing for more flexibility in the choice of coupling parameters and reducing unwanted dephasing due to residual resonator photon population^30^. A simplified circuit diagram of the device is shown in Fig. 1f.

The superconducting qubit is of transmon type and consists of a single superconducting aluminum (Al) island shunted to ground via a SQUID (orange in Fig. 1). The transmon charging and Josephson energies are *E*_c_/*h* ~243.0 ± 0.2 MHz and $E_{\mathrm{J}}^{0}/h\sim 30.1 \pm 0.1\,{\mathrm{GHz}}$, respectively (see Supplementary Note 2 for more information). The transition frequency *ω*_tr_ between its ground state |*g*〉 and excited state |*e*〉 is adjusted by using the magnetic flux generated in the transmon SQUID loop by a flux line (purple in Fig. 1). We read out the state of the transmon qubit with a 50 Ω coplanar waveguide resonator (dark blue in Fig. 1) capacitively coupled to the qubit^14,31^.

The DQD charge qubit (Fig. 1d), schematically indicated by the two light blue dots in Fig. 1f, is defined by standard depletion gate technology using Al top gates on a GaAs/AlGaAs heterostructure that hosts a two-dimensional electron gas (2DEG)^15,21,32^. The DQD is tuned to the few-electron regime and its excitation energy is given by $\omega _{{\mathrm{DQD}}} = \sqrt {4t_{\mathrm{c}}^2 + \delta ^2}$, with the inter-dot tunnel rate *t*_c_ and the DQD energy detuning *δ*.

We use a superconducting high-impedance resonator for mediating interactions between the transmon and the DQD^21^. The resonator is composed of an array of 35 SQUIDs (Fig. 1b), and is capacitively coupled to both transmon and DQD charge qubits (see Fig. 1b, f). It is grounded at one end and terminated in a small island at the other end to which a single coplanar drive line is capacitively coupled (green in Fig. 1b, c). A gate line extends from the island and forms one of the plunger gates of the DQD (in red in Fig. 1d)^21,32^. The high impedance of the resonator increases the strength of the vacuum fluctuations of electric field, enhancing the coupling strength of the individual qubits to the resonator (see Supplementary Note 2 for more information).

We characterize the hybrid circuit by measuring the amplitude and phase change of the reflection coefficient of a coherent tone at frequency *ω*_p_ reflected from the multiplexed resonators (the microwave setup is presented in Supplementary Fig. 1). The response changes with the potentials applied to the gate electrodes forming the DQD and the magnetic flux applied to the transmon. By varying the DQD detuning *δ* and the transmon flux Φ_tr_, each qubit is individually tuned into resonance with the high-impedance resonator. The coupling strengths measured between the SQUID array resonator and the DQD charge qubit and the transmon qubit are 2*g*_DQD,Sq_/2*π* ~66.2 ± 0.4 MHz (at *ω*_r,Sq_/2*π* = 4.089 GHz) and 2*g*_tr,Sq_/2*π* ~451.3 ± 0.3 MHz (at *ω*_r,Sq_/2*π* = 5.180 GHz), respectively, for more details see Supplementary Note 5 and Supplementary Fig. 3. For the same configuration, we extract the linewidth of the qubits spectroscopically^21,30^ and find *δω*_DQD_/2*π* ~2.7 ± 0.4 MHz and *δω*_tr_/2*π* ~0.78 ± 0.05 MHz. Both subsystems individually are in the strong coupling regime (2*g* > *κ*/2 + *γ*_2_) with a SQUID array resonator linewidth of *κ*/2*π* = (*κ*_ext_ + *κ*_int_)/2*π* ~(3 + 5) MHz.

### Resonant interaction

**Fig. 2:**
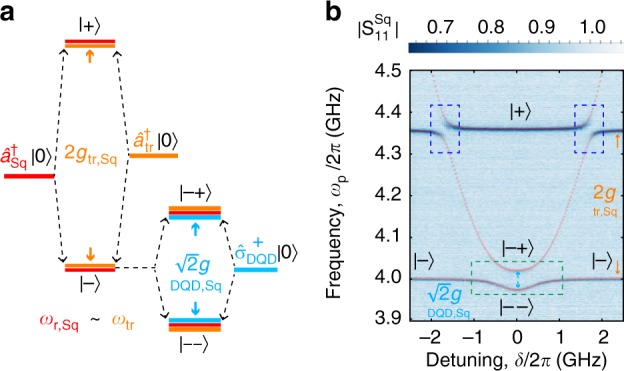
Resonant interaction between the DQD charge qubit, the SQUID array resonator and the transmon. **a** Energy level diagram of the DQD-SQUID array-transmon system for the bias point considered in panel (**b**). The energy levels are colored in accordance with the code used in Fig. 1. **b** Reflectance $|S_{11}^{{\mathrm{Sq}}}|$ of the SQUID array resonator hybridized with the transmon and DQD as a function of the DQD detuning *δ* at the bias point discussed in the main text. Red dots are obtained by numerical diagonalization of the system Hamiltonian [see Eq. (1) in Supplementary Note 2], using parameters extracted from independent spectroscopy measurements

To demonstrate the coherent coupling between the transmon qubit and the DQD charge qubit, we first characterize the configuration with the three systems interacting resonantly with each other (see Fig. 2). We tune the SQUID array into resonance with the transmon and observe the vacuum Rabi modes $| \mp \rangle = \left( {{\mathrm{sin}}\theta _{\mathrm{m}}\hat a_{{\mathrm{Sq}}}^\dagger \pm {\mathrm{cos}}\theta _{\mathrm{m}}\hat a_{{\mathrm{tr}}}^\dagger } \right)|0\rangle$, with the ground state of the system |0〉 = |0〉_Sq_ ⊗ |*g*〉_tr_ ⊗ |*g*〉_DQD_ and the creation operators for the excitations in the SQUID array (transmon) $\hat a_{{\mathrm{Sq}}}^\dagger$ ($\hat a_{{\mathrm{tr}}}^\dagger$). The mixing angle *θ*_m_ is determined by tan2*θ*_m_ = 2*g*_tr,Sq_/|Δ_tr_|, with $|\Delta _{{\mathrm{tr}}}| = |\omega _{{\mathrm{tr}}}^\prime - \omega _{{\mathrm{r,Sq}}}|$ and the transmon excitation frequency $\omega _{{\mathrm{tr}}}^\prime$ dressed by the interaction with the 50 Ω resonator. We then configure the DQD electrostatic gate voltages to tune its transition frequency at the charge sweet spot [*ω*_DQD_(*δ* = 0) = +2*t*_c_] into resonance with the lower transmon-SQUID array Rabi mode |−〉. From the hybridization between the states |−〉 and the DQD excited state $\hat \sigma _{{\mathrm{DQD}}}^ + |0\rangle$, we obtain the states |−−〉 and |−+〉, leading to the avoided crossing enclosed by the green dashed box in Fig. 2b. Similarly, when the DQD excitation energy is equal to the energy of the higher transmon-SQUID array Rabi mode, |+〉, the hybrid system develops two avoided crossings at the respective detunings *δ* in the spectrum (see blue dashed box in Fig. 2b). The observed spectrum resulting from the hybridization of the three quantum systems is in good agreement with our calculation (see red dots in Fig. 2b and “System Hamiltonian” section in Supplementary Note 2 for more information).

### Dispersive interaction

Next, we discuss the virtual photon-mediated coherent interaction between the DQD and the transmon qubit. This is realized in the dispersive regime, where both qubit frequencies are detuned from the high-impedance resonator. In this regime, no energy is exchanged between the qubits and the resonator, and the strength of the effective coherent interaction between the two qubits is given by 2*J* ~*g*_tr,Sq_*g*_DQD,Sq_/(1/|Δ_tr_| + 1/|Δ_DQD_|)^19,20^. We spectroscopically explore this qubit–qubit coupling by applying a probe tone at frequency *ω*_p_/2*π* = 6.5064 GHz to the transmon readout resonator. The reflectance of the probe tone from the 50 Ω resonator is measured while a microwave spectroscopy tone of frequency *ω*_s_ is swept across the transmon transition frequency to probe its excitation spectrum^30^.

**Fig. 3:**
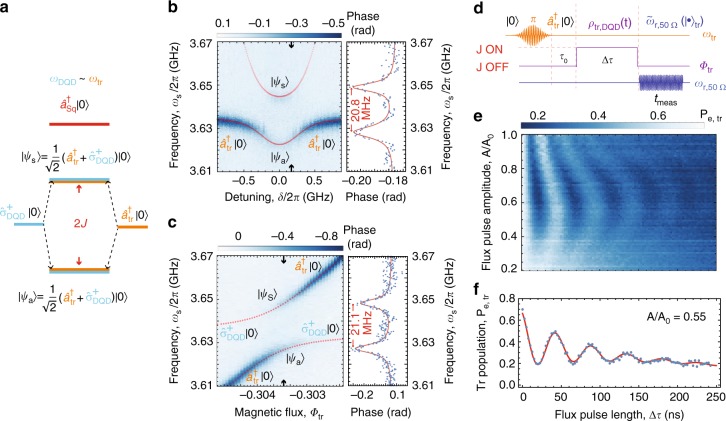
DQD-transmon interaction mediated by virtual photon exchange in the SQUID array resonator. **a** Energy level diagram of the DQD-transmon qubit coupling mediated via dispersive interaction with the SQUID array resonator (red line). The DQD excitation $(\sigma _{{\mathrm{DQD}}}^\dagger |0\rangle )$, the transmon excitation $(a_{{\mathrm{tr}}}^\dagger |0\rangle )$ and the SQUID array resonator excitation $(a_{{\mathrm{Sq}}}^\dagger |0\rangle )$ are shown, together with their hybridized states |Ψ_s,a_〉 and the system vacuum state |0〉 = |0〉_Sq_ ⊗ |*g*〉_tr_ ⊗ |*g*〉_DQD_. **b** Left: spectroscopy of the DQD qubit interacting with the transmon. Phase Δ*ϕ* = Arg[*S*_11_] of a fixed frequency measurement tone *ω*_p_/2*π* = 6.5064 GHz = *ω*_r,50Ω_/2*π* reflected off the 50 Ω CPW read-out resonator vs. transmon qubit spectroscopy frequency *ω*_s_ and DQD qubit detuning *δ* [(**c**) the flux through the SQUID loop of the transmon Φ_tr_]. Right: phase Δ*ϕ* = Arg[*S*_11_] response at the DQD detuning *δ* [(c) at the flux Φ_tr_] indicated by the black arrows in left panel showing a coupling splitting of 2*J* ~20.8 ± 0.3 MHz [2*J* ~21.1 ± 0.2 MHz]. **d** Pulse protocol for the population transfer between the transmon and the DQD charge qubit. *ρ*(*t*) indicates the density matrix of the coupled transmon-DQD system during the interaction time Δ*τ* and $\tilde \omega _{{\mathrm{r,50\Omega }}} = \omega _{{\mathrm{r,50\Omega }}} + g_{{\mathrm{tr,50\Omega }}}^2/{\mathrm{\Delta }}_{{\mathrm{tr,50\Omega }}}$. *τ*_0_ is a finite time difference between the preparation pulse and the flux pulse. **e** Average transmon excited state population *P*_e,tr_ (each data point is the intergrated average over 50,000 repetitions of the experiment), as a function of the flux pulse length Δ*τ* and normalized flux pulse amplitude *A*/*A*_0_. **f** Transmon excited state population *P*_e,tr_ vs. Δ*τ* for a flux pulse amplitude of *A*/*A*_0_ = 0.55, for which the transmon is approximately in resonance with the DQD (*ω*_tr_/2*π* ~*ω*_DQD_/2*π*=3.660 GHz). *ω*_r,Sq_/2*π* = 4.060GHz and *ω*_r,50Ω_/2*π* = 6.5048 GHz. The red line is a fit to a Markovian master equation model (see Supplementary Note 6 for more details)

To observe the coherent DQD-transmon coupling, we either tune *δ* to bring the DQD into resonance with the transmon (see Fig. 3b) or tune Φ_tr_ to bring the transmon into resonance with the DQD (see Fig. 3c). In either case, when the qubit frequencies are in resonance, as depicted in Fig. 3a, a clear avoided crossing of magnitude 2*J*/2*π* ~21.1 ± 0.2 MHz (Fig. 3c), larger than the combined linewidth of the coupled system (*γ*_DQD_ + *γ*_tr_)/2*π* ~4.6 ± 0.5 MHz, is observed. The observed resonance frequencies are in good agreement with our simulation (red dots in Fig. 3b, c) for the explored configuration characterized by |Δ_DQD_| ~10*g*_DQD,Sq_ and |Δ_tr_| ~3*g*_tr,Sq_. The well-resolved DQD-transmon avoided crossing demonstrates that the high-impedance resonator mediates the coupling between the semiconductor and the superconducting qubit.

### Time-resolved population transfer oscillations

We demonstrate virtual-photon mediated coherent population transfer between the transmon and DQD charge qubits in time-resolved measurements. We induce the exchange coupling by keeping the DQD and SQUID array cavity frequencies fixed at *ω*_DQD_(*δ* = 0)/2*π* = 3.66 GHz and *ω*_r,Sq_/2*π* = 4.06 GHz, respectively, and varying the transmon frequency nonadiabatically^18,19^ using the pulse protocol illustrated in Fig. 3d. Initially, both qubits are in their ground state and the effective coupling between them is negligible, due to the large difference between their excitation frequencies. Next, we apply a *π*-pulse to the transmon qubit to prepare it in its excited state $a_{{\mathrm{tr}}}^\dagger |0\rangle$. Then, a nonadiabatic current pulse, applied to the flux line, changes the flux Φ_tr_ and tunes the transmon into resonance with the DQD charge qubit for a time Δ*τ*, which we vary between 0 and 250 ns. After the completion of the flux pulse controlling the interaction, the state of the transmon is measured (for a time *t*_meas_) through its dispersive interaction with the 50 Ω CPW resonator. We observe coherent oscillations of the transmon excited state population as a function of the interaction time Δ*τ* (see Fig. 3e, f).

The population oscillations during the time Δ*τ* are caused by the exchange interaction with coupling strength *J*(|Δ_tr_|, |Δ_DQD_|). Oscillations in both the transmon population and in the DQD occur as the excitation is transferred between the two, Fig. 3e. Due to energy relaxation and dephasing of both systems, the population oscillations are damped and the mean population decays over time. In the current experiment, we were able to only extract the transmon population. Future experiments may allow to also measure the DQD population and to explore the correlations between the two qubits to explicitly verify the entanglement of the two systems. As a result, we observe the characteristic chevron pattern in the transmon qubit population in dependence on the flux pulse amplitude and length (see Fig. 3e)^18^.

A trace of the population oscillation pattern at fixed pulse amplitude $A/A_0\sim 0.55$, approximately realizing the DQD-transmon resonance condition ($\omega _{{\mathrm{tr}}}^\prime /2\pi = \omega _{{\mathrm{DQD}}}/2\pi = 3.66\,{\mathrm{GHz}}$), is in excellent agreement with the Markovian master equation simulation (see the red line in Fig. 3f and Supplementary Note 6 for more information). The simulations are performed within the dispersive approximation in which the qubits interact with rate 2*J*/2*π* = 21.8 ± 0.1 MHz, via an exchange interaction consistent with the spectroscopically measured energy splitting 2*J*/2*π* ~21.1 ± 0.2 MHz (Fig. 3c).

## Discussion

Further improvements in device design and sample parameters could allow to increase the visibility and fidelity of the coherent population transfer protocol between the two different quantum computing platforms. By increasing the bus resonator impedance even further, e.g., by making use of a Josephson junction array (instead of a SQUID array), it will be possible to increase the coupling strength of the artificial atoms with the radiation field by about a factor of two. This will allow also to increase the frequency detuning between the qubits and the quantum bus, which will result in a reduction of the relaxation rate of the transmon, limited by Purcell decay in the current experiment. This can be also achieved by reducing the *k*_tot_ of the bus resonator or realizing a Purcell filter^33^. Furthermore, the realization of the superconducting elements on a more suitable substrate, such as sapphire or silicon, could allow to further reduce the intrinsic loss of the transmon and of the high impedance resonator while maintaining the possibility to capacitively couple to the DQD structure, making use of flip-chip technology^34^. From the semiconductor qubit side, moving to silicon as host material could allow to reach a longer coherence time for both the charge and the spin degrees of freedom of the confined electron.

In this work, we realized an interface between semiconductor- and superconductor-based qubits by exchanging virtual photons between two distinct physical systems in a hybrid circuit QED architecture^35,36^. The coherent interaction between the qubits is witnessed both by measurements of well-resolved spectroscopic level splitting and by time-resolved population oscillations. The interaction can be enabled both electrically via the QD and magnetically via the transmon qubit. The resonator mediated coupling also provides for nonlocal coupling to the semiconductor qubit, demonstrated here over distances of more than 50 μm. We expect the approach demonstrated here for the charge degree of freedom of a semiconductor qubit to be transferable to the spin degree of freedom and also to other material systems such as Si or SiGe^37–39^. In this way, the coupling to electron spin or even nuclear spin qubits may provide an avenue for realizing a spin based quantum memory, which can be interfaced to other solid state qubits, including superconducting ones. In addition, the combination of short distance coupling and control in semiconductor qubits with long-distance coupling through microwave resonators provided by circuit QED may indicate a viable solution to the wiring and coupling challenge in semiconductor qubits^40^ and may be essential for realizing error correction in these systems, for example by using the surface code^41^.

## Supplementary information

Supplementary Information

## Data Availability

The authors declare that the data supporting the findings of this letter and corresponding Supplementary Information file are available online at the ETH Zurich repository for research data https://doi.org/10.3929/ethz-b-000347083.
