# Supplementary material for: Coherent microwave-photon-mediated coupling between a semiconductor and a superconducting qubit
Source: Nat Commun. 2019 Jul 8;10:3011. doi: 10.1038/s41467-019-10798-6 (PMC6614454; doi:10.1038/s41467-019-10798-6)
Supplement: Supplementary file 1 — Supplementary Information [file 41467_2019_10798_MOESM1_ESM.pdf]

Supplementary Information for the Article:

Coherent microwave-photon-mediated coupling between a semiconductor and a superconducting qubit

by P. Scarlino *et al.*

SUPPLEMENTARY NOTE 1

Device and measurement setup

The device is realized on a $GaAs/Al_xGa_{1-x}As$ heterostructure. The 2DEG, embedded 90 nm below the surface at the interface of $GaAs/Al_xGa_{1-x}As$, has been removed by etching everywhere but in a small region hosting the DQD [see Figs. 1(a,b)]. The gate structures that define the DQD confinement potential are realized using a combination of gold (Au) top gates for the coarse gate structures [yellow in Figs. 1(a-c)], and aluminum (Al) for the fine gate structures [light gray in Figs. 1(a,b)]. The tunnel junctions of the SQUIDs are formed by two Al electrodes separated by a thin oxide layer. They are fabricated using standard electron-beam lithography and shadow evaporation of 30 nm and 120 nm aluminum (with in-situ oxidation).

We characterize the hybrid circuit by measuring the amplitude and phase change of the reflection coefficient of a coherent tone reflected at frequency ω_p off the multiplexed SQUID array and $50\,\Omega$ CPW resonators (see Supplementary Figure 1). The multiplexing of the two resonators is realized by cascading two circulators and connecting the reflection port of each resonator to a circulator (see Supplementary Figure1). The microwave tone is generated at room-temperature and is attenuated by -20 dB at the 4 K, 100 mK and 20 mK stages before passing through a circulator which routes it to the resonator and routes the reflected signal to the output line. In the output line the reflected signal is amplified using a cryogenic HEMT (+39 dB) at 4 K and by two amplifiers (+33 dB each) at room-temperature, before it is down converted to an intermediated frequency (IF) of 250 MHz. With +29 dB amplification the IF is acquired at 1Gs/s using an Acqiris U1084A PCIe 8-bit High-Speed Digitizer. The DC voltages are supplied to the gates by Yokogawa 7651 DC programmable sources with a 1:10 voltage divider also acting as a low pass filter (1 Hz cut-off). The source and drain of the DQD were grounded in the experiment. At base temperature, 2-stage RC filters with 160 kHz and 16 kHz cut-off are used at the input of shielded lines leading to the sample holder. A schematic of the complete setup with all important components is displayed in Supplementary Figure 1. The experiment is performed at a cryostat temperature of 30 mK.

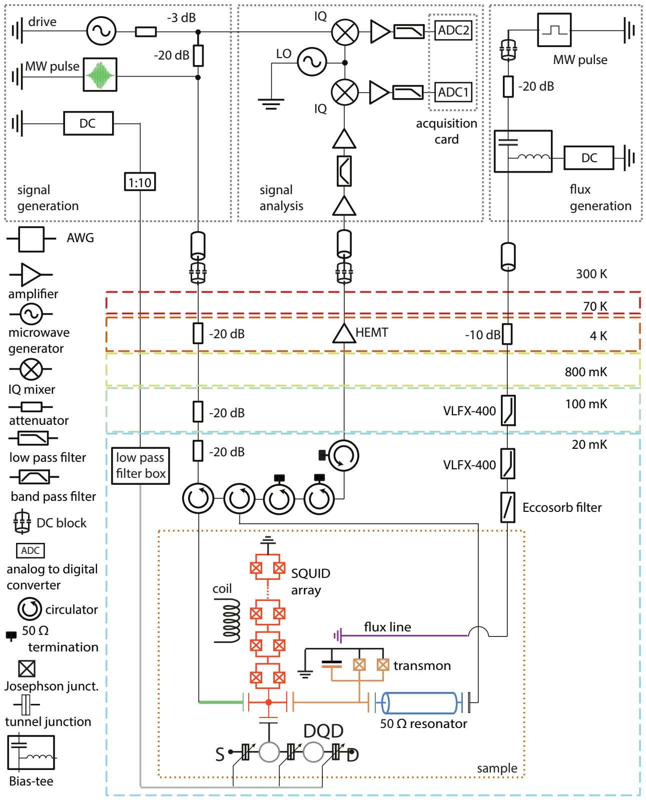

Supplementary Figure 1. Simplified schematic of cryogenic and room-temperature measurement setup, further details in text.

SUPPLEMENTARY NOTE 2

System Hamiltonian

The coupled quantum system is described by the Hamiltonian

$$\begin{aligned}\hat{H}_{\text{tot}} =& \hat{H}_{\text{DQD}} + \hat{H}_{\text{tr}} + \hat{H}_{\text{r, Sq}} + \hat{H}_{\text{r, 50}\,\Omega} + \\ &+ \hat{H}_{\text{DQD, Sq}} + \hat{H}_{\text{tr, Sq}} + \hat{H}_{\text{tr, 50}\,\Omega},\end{aligned} \tag{1}$$

$$\hat{H}_{\text{DQD}} = \frac{\omega_{\text{DQD}}(t_{\text{c}}, \delta)}{2}\sigma^z_{\text{DQD}},\ \hat{H}_{\text{tr}} = \sum_{i=1}^{n_{\text{tr}}} \omega_{i,\text{tr}}(\Phi_{\text{Sq}}, \Phi_{\text{tr}})|i\rangle\langle i|, \tag{2}$$

$$\hat{H}_{\text{r, Sq}} = \omega_{\text{r,Sq}}(\Phi_{\text{Sq}})\hat{a}^\dagger_{\text{Sq}}\hat{a}_{\text{Sq}},\ \hat{H}_{\text{r, 50}\,\Omega} = \omega_{\text{r,50}\Omega}\hat{b}^\dagger\hat{b}, \tag{3}$$

$$\hat{H}_{\text{DQD, Sq}} = g_{\text{DQD,Sq}}(\Phi_{\text{Sq}}, t_{\text{c}}, \delta)(\sigma^-_{\text{DQD}}\hat{a}^\dagger_{\text{Sq}} + \sigma^+_{\text{DQD}}\hat{a}_{\text{Sq}}), \tag{4}$$

$$\hat{H}_{\text{tr, Sq}} = \sum_{i,j=1}^{n_{\text{tr}}} g_{\text{tr,Sq}}(\Phi_{\text{Sq}}, \Phi_{\text{tr}})n_{i,j}|i\rangle\langle j|(\hat{a}^\dagger_{\text{Sq}} + \hat{a}_{\text{Sq}}), \tag{5}$$

$$\hat{H}_{\text{tr, 50}\Omega} = \sum_{i,j=1}^{n_{\text{tr}}} g_{\text{tr,50}\Omega}(\Phi_{\text{Sq}}, \Phi_{\text{tr}})n_{i,j}|i\rangle\langle j|(\hat{b}^\dagger + \hat{b}), \tag{6}$$

with $\hbar = 1$, and $\hat{a}_{\text{Sq}}$, $\hat{b}$ and $\hat{\sigma}^-_{\text{DQD}}$ are the annihilation operator for the excitations of the SQUID array, $50\,\Omega$ resonator and lowering operator of the DQD qubit, respectively. $\omega_{\text{r,Sq}}$ and $\omega_{r,50\Omega}$ are the resonance frequency of the SQUID array and of $50\,\Omega$ CPW resonator, respectively. The DQD charge qubit energy is given by $\omega_{\text{DQD}} = \sqrt{4t_{\text{c}}^2 + \delta^2}$, where t_{c} and δ are the inter-dot tunnel rate and DQD energy detuning, respectively. $\omega_{i,\text{tr}}(\Phi_{\text{tr}})$ and $|i\rangle$ are the frequency and state of the i-level of the transmon, respectively. $n_{i,j} = \langle i|\hat{n}|j\rangle$ are the Cooper pair number matrix elements, and n_{tr} is the number of levels forming the transmon qubit (in our model $n_{\text{tr}} = 4$) [1]. The coupling strengths between the transmon-SQUID array, transmon-50Ω resonator, and DQD-SQUID array are indicated with $g_{\text{tr,Sq}}(\Phi_{\text{Sq}}, \Phi_{\text{tr}})$, $g_{\text{tr,50}\Omega}(\Phi_{\text{tr}})$ and $g_{\text{DQD,Sq}}(\Phi_{\text{Sq}}, t_{\text{c}}, \delta)$, where Φ_{Sq} and Φ_{tr} are the external magnetic fluxes through the SQUID loops of the resonator array (assumed uniformly threaded) and transmon, respectively.

SQUID array resonator

The SQUID array resonator is formed by $N_{\text{Sq}} = 35$ SQUIDs in series with extra $N_{\text{sj}} = 34$ single junctions generated during the shadow evaporation process. The total inductance is

$$L(\Phi_{\text{Sq}}) = N_{\text{Sq}}L_{\text{Sq}}\left(\beta + \frac{1}{|\cos(\Phi'_{\text{Sq}}/\Phi_0)|}\right), \tag{7}$$

with the magnetic flux quantum Φ_0, and the total magnetic flux through the SQUID array $\Phi'_{\text{sq}} = 2\pi\gamma\Phi_{\text{Sq}} + 2\pi\Phi_c$. Here, γ and Φ_c are constants that were parameterized by fitting Supplementary Figure 2(b). $\beta = N_{\text{sj}}L_{\text{sj}}/N_{\text{Sq}}L_{\text{Sq}}$ is a parameter that takes into account the presence of an extra constant inductive contribution, in series with the SQUID array inductance, generated during the shadow mask deposition process [2, 3]. In our experiment $\beta \sim 0.1$. In a lumped element model, the SQUID array frequency is

$$\omega_{\text{r,Sq}}(\Phi_{\text{Sq}}) = \frac{\omega^0_{\text{r,Sq}}}{\left(\beta + \frac{1}{|\cos(\Phi'_{\text{Sq}}/\Phi_0)|}\right)^{1/2}}, \tag{8}$$

with $\omega^0_{\text{r,Sq}} = 1/\sqrt{CN_{\text{Sq}}L_{\text{Sq}}}$ and C an effective capacitance that takes into account the capacitance of the SQUID array to the ground and to the transmon.

Transmon qubit

The transmon qubit in our device is formed by a single island capacitor shunted to ground by two Josephson junctions in a SQUID geometry. We control the qubit frequency by an external magnetic flux Φ_{tr}. For symmetric junctions, the Josephson

energy is $E_{\rm J}(\Phi_{\rm Sq},\Phi_{\rm tr}) = E_{\rm J}^0|\cos(\Phi'_{\rm tr}/\Phi_0)|$, with the Josephson energy at zero flux E_J^0, the total magnetic flux through the transmon $\Phi'_{\rm tr} = 2\pi\alpha\Phi_{\rm Sq} + 2\pi\Phi_{\rm tr}$. It depends on both external fluxes $\Phi_{\rm tr}$ and $\Phi_{\rm Sq}$. Here, α is the ratio between the areas of the SQUID loops of the transmon and the SQUID array resonator. The transmon Hamiltonian can be written as

$$\hat{H}_{\rm tr} = 4E_c(\hat{n}-n_g)^2 - E_{\rm J}(\Phi_{\rm Sq},\Phi_{\rm tr})\cos\hat{\varphi}, \tag{9}$$

with the Cooper pair number operator $\hat{n}$, the effective offset charge n_g of the device, the phase difference $\hat{\varphi}$ across the junction, and the transmon charging energy E_c. We approximate the transmon frequency (0 to 1 transition) as [1]

$$\omega_{\rm tr}(\Phi_{\rm Sq},\Phi_{\rm tr}) = \omega_{\rm pl,tr}|\cos(\Phi'_{\rm tr}/\Phi_0)|^{1/2} - E_c \tag{10}$$

with the plasma frequency $\omega_{\rm pl,tr} = \sqrt{8E_cE_J^0}$ obtained by fitting the model to the data in Supplementary Figure 2. Finally, the transmon Hamiltonian is diagonalized numerically considering four states.

Double quantum dot qubit

The Hamiltonian describing the DQD charge qubit is

$$\hat{H}_{\rm DQD} = \frac{\delta}{2}\hat{\tau}_z + t_c\hat{\tau}_x \tag{11}$$

where δ is the detuning between the two dots, t_c is the interdot tunneling coupling, and $\hat{\tau}_i$ are the three Pauli matrices defined in the $|L\rangle$ and $|R\rangle$ basis, the bases for a single charge to be either on the left or the right QD [$\hat{\tau}_z = |R\rangle\langle R| - |L\rangle\langle L|$].

A basis rotation is performed to diagonalize the DQD Hamiltonian, resulting in

$$\hat{H}_{\rm DQD} = \frac{\omega_{\rm DQD}}{2}\sigma^z_{\rm DQD}, \tag{12}$$

where $\omega_{\rm DQD} = \sqrt{\delta^2+4t_c^2}$ and $\sigma^z_{\rm DQD} = |+\rangle_{\rm DQD}\langle+|_{\rm DQD} - |-\rangle_{\rm DQD}\langle-|_{\rm DQD}$, with

$$|+\rangle_{\rm DQD} = \cos(\theta/2)|R\rangle - \sin(\theta/2)|L\rangle \tag{13}$$
$$|-\rangle_{\rm DQD} = \sin(\theta/2)|R\rangle + \cos(\theta/2)|L\rangle \tag{14}$$

and $\tan\theta = 2t_c/\delta$.

SUPPLEMENTARY NOTE 3

Description of the strategy used to get the parameters for the modelling

The coupling strengths between the transmon and SQUID array, the transmon and 50 Ω resonator, and the DQD and SQUID array are defined as:

$$g_{\rm tr,Sq}(\Phi_{\rm Sq},\Phi_{\rm tr}) = g^0_{\rm tr,Sq}\frac{|\cos(\Phi'_{\rm tr}/\Phi_0)|^{1/4}}{\left(\beta + \frac{1}{|\cos(\Phi'_{\rm Sq}/\Phi_0)|}\right)^{1/4}}, \tag{15}$$

$$g_{\rm tr,50\ \Omega}(\Phi_{\rm Sq},\Phi_{\rm tr}) = g^0_{\rm tr,50\Omega}|\cos(\Phi'_{\rm tr}/\Phi_0)|^{1/4}, \tag{16}$$

$$g_{\rm DQD,Sq}(\Phi_{\rm Sq},t_c,\delta) = \frac{g^0_{\rm DQD,Sq}}{\left(\beta + \frac{1}{|\cos(\Phi'_{\rm Sq}/\Phi_0)|}\right)^{1/4}}\frac{2t_{\rm c}}{\omega_{\rm DQD}(t_{\rm c},\delta)}. \tag{17}$$

Here, $\Phi'_{\rm Sq}$ and $\Phi'_{\rm tr}$ are the external (total) magnetic flux through the SQUID loops of the resonator array and the transmon. The term $2t_{\rm c}/\omega_{\rm DQD}$ in Eq. (17) corresponds to the mixing angle renormalization of the DQD-resonator interaction strength [4].

The parameters $g^0_{\rm tr,Sq}$ and $g^0_{\rm tr,50\Omega}$ are obtained by fitting the experimental data in Supplementary Figure 2 (more details are presented in the following section). The DQD-SQUID array coupling in Eq. (17) is obtained by considering $g_{\rm DQD,Sq}(\Phi_{\rm Sq}) \propto Z_{\rm Sq}^{-1/2}(\Phi_{\rm Sq})$, where $Z_{\rm Sq}(\Phi_{\rm Sq}) = \sqrt{L(\Phi_{\rm Sq})/C}$ is the SQUID array impedance. $g^0_{\rm DQD,Sq}$ is obtained by fitting the vacuum Rabi splitting data presented in Supplementary Figure 3 with the other parameters fixed.

We calculate the spectrum of the DQD-SQUID array-transmon system by numerical diagonalization of the Hamiltonian (1) using parameters extracted from independent spectroscopy measurements. The parameters used to obtain theory points in Fig. 2 and Fig. 3 are listed in the Supplementary Tables 1, 2 and 3.

$2t_c$	3993 MHz
$\omega_{tr}/2\pi$	4150 MHz
$\omega_{r,Sq}/2\pi$	4230 MHz
$g_{tr,Sq}/2\pi$	166 MHz
$g_{DQD,Sq}/2\pi$	34 MHz
$g_{tr,50\Omega}/2\pi$	98 MHz

Supplementary Table 1. Parameters used to obtain the calculated eigenenergies shown in Fig. 2(b).

$2t_c$	3635 MHz
$\omega_{tr}/2\pi$	3695 MHz
$\omega_{r,Sq}/2\pi$	4062 MHz
$g_{tr,Sq}/2\pi$	128 MHz
$g_{DQD,Sq}/2\pi$	36 MHz
$g_{tr,50\Omega}/2\pi$	93 MHz

Supplementary Table 2. Parameters used to obtain the calculated eigenenergies shown in Fig. 3(b).

$2t_c$	3638 MHz
$\omega_{tr}/2\pi$	3695 MHz
$\omega_{r,Sq}/2\pi$	4062 MHz
$g_{tr,Sq}/2\pi$	128 MHz
$g_{DQD,Sq}/2\pi$	36 MHz
$g_{tr,50\Omega}/2\pi$	93 MHz

Supplementary Table 3. Parameters used to obtain the calculated eigenenergies shown in Fig. 3(c).

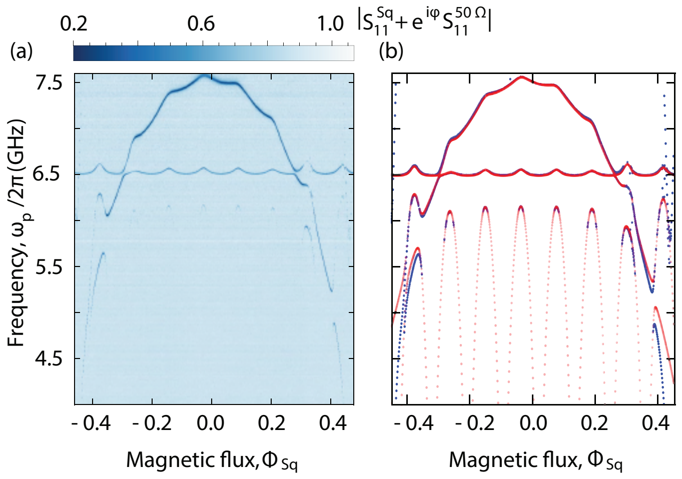

Supplementary Figure 2. Flux tunability of the SQUID array and Transmon qubit. (a) Reflectance spectrum $|S_{11}^{Sq}(\omega_p) + e^{i\varphi} S_{11}^{50\Omega}(\omega_p)|$ (with φ the phase accumulated by the microwave signal in between the two resonators) of the multiplexed SQUID array and 50 Ω CPW resonators as a function of probe frequency $\omega_p/2\pi$ and applied magnetic flux Φ_{Sq} (expressed in flux quanta for the periodicity of the SQUID array) via the coil. (b) Resonance frequencies extracted from the dips in the $|S_{11}^{Sq}(\omega_p) + e^{i\varphi} S_{11}^{50\Omega}(\omega_p)|$ (blue points) and simulated spectrum (red points) of the transmon interacting with the SQUID array and the 50 Ω CPW resonators, according to Eq. (1) with parameters $\omega_{pl,tr}/2\pi = 6.550$ GHz, $E_c/h = 243$ MHz, $\omega^0_{r,Sq}/2\pi = 7.867$ GHz, $\omega_{r,50\Omega}/2\pi = 6.490$ GHz, $\beta = 0.1$, $g^0_{tr,Sq}/2\pi = 230$ MHz, $g^0_{tr,50\Omega}/2\pi = 120$ MHz.

SUPPLEMENTARY NOTE 4

Interaction between the transmon qubit and the SQUID array and 50 Ω resonators

We analyse the flux dependence of the system transition frequencies to fix the parameters $\omega^0_{\mathrm{r,Sq}}$, $\omega_{\mathrm{r,50\Omega}}$, $\omega_{\mathrm{pl,tr}}$, $g^0_{\mathrm{tr,Sq}}$, $g^0_{\mathrm{tr,50\Omega}}$ for the modelling of the spectroscopy measurements by measuring the reflectance of the multiplexed SQUID array and redout resonator as a function of SQUID array flux Φ_{Sq}, with the DQD far detuned from the SQUID array resonator (Supplementary Figure 2). The blue dots in Supplementary Figure 2(b) are experimentally extracted resonance frequencies from Supplementary Figure 2(a). The red dots are the eigenvalues of the calculated system Hamiltonian (1), obtained by making use of the following parameters: $\omega_{\mathrm{pl,tr}}/2\pi = 6.550$ GHz, $\omega^0_{\mathrm{r,Sq}}/2\pi = 7.867$ GHz, $\omega_{\mathrm{r,50\Omega}}/2\pi = 6.490$ GHz, $E_{\mathrm{c}}/h = 243$ MHz, $\beta = 0.1$, $g^0_{\mathrm{tr,Sq}}/2\pi = 230$ MHz, $g^0_{\mathrm{tr,50\Omega}}/2\pi = 120$ MHz. We parameterize the total magnetic flux of the SQUID array as: $\Phi'_{\mathrm{sq}} = 2\pi\gamma\Phi_{\mathrm{Sq}} + 2\pi\Phi_{\mathrm{c}}$, with $\gamma \sim 0.43$ and $\Phi_{\mathrm{c}} \sim 0.0072 \times \Phi_0$. The transmon magnetic flux is $\Phi'_{\mathrm{tr}} = 2\pi\alpha\Phi_{\mathrm{Sq}} + 2\pi\Phi_{\mathrm{tr}}$, with the ratio $\alpha \sim 4.41$ between the areas of the SQUID loops of the transmon and the SQUID array resonator. To fit the data points in Supplementary Figure 2, we fixed $\Phi_{\mathrm{tr}} \sim 0.162 \times \Phi_0$.

We note that the transmon and 50 Ω resonator are well described by the model for all values of applied flux Φ_{Sq}. The dependence of the SQUID array resonant frequency from Φ_{Sq} is well captured for $-0.3 \leq \Phi_{\mathrm{Sq}}/\Phi_0 \leq 0.3$. However, for $|\Phi_{\mathrm{Sq}}| > 0.3 \times \Phi_0$ the lumped element description fails to describe the the SQUID array cavity properties accurately. One reason might be due to non-linearity of the Josephson junctions forming the SQUID array resonator, or to inhomogeneity in the magnetic flux threading the SQUIDs of the array. The parameters of the 50 Ω resonator and transmon are kept fixed for all the other fits. We adjust only parameters related to the SQUID array and DQD, according to the expressions in Eqs. (8), (10), (15)-(17).

SUPPLEMENTARY NOTE 5

Coherent coupling of DQD to SQUID array and transmon to SQUID array

To quantify the coupling strength between the SQUID array resonator and the DQD, we first fix the transmon resonance frequency $\omega_{\mathrm{tr}}/2\pi < 2\,\mathrm{GHz}$, the SQUID array resonance frequency $\omega_{\mathrm{r,Sq}}/2\pi = 4.089\,\mathrm{GHz}$ and adjust the tunnel coupling of the DQD to the same frequency [see Supplementary Figure 3(a)].

With the DQD far detuned, the undercoupled ($\kappa_{\mathrm{int}} > \kappa_{\mathrm{ext}}$) SQUID array resonator displays the bare linewidth $\kappa/2\pi = (\kappa_{\mathrm{ext}}+\kappa_{\mathrm{int}})/2\pi \sim (4{+}8)$ MHz. Varying the DQD detuning δ, we bring the DQD into resonance with the SQUID array resonator ($\omega_{\mathrm{r,Sq}} = \omega_{\mathrm{DQD}}$) close to the DQD sweetspot, indicated by arrows in Supplementary Figure 3(a). We observe a clear vacuum Rabi mode splitting in the reflectance spectrum of the resonator [Supplementary Figure 3(b)]. A fit (red line) of the spectrum to two Lorentzian lines yields a splitting of $2g_{\mathrm{DQD,Sq}}/2\pi \sim 66.2 \pm 0.4$ MHz, with an effective linewidth $\delta\omega_{\mathrm{DQD}}/2\pi \sim 7.1 \pm 1.1$ MHz dominated by the loss of the SQUID array resonator. Keeping the DQD in Coulomb blockade ($\omega_{\mathrm{DQD}}/2\pi > 10\,\mathrm{GHz}$), we tune the transmon frequency ω_{tr} while keeping the SQUID array resonance frequency fixed at $\omega_{\mathrm{r,Sq}}/2\pi = 5.1813\,\mathrm{GHz}$, by using a linear combination of the magnetic flux generated by a superconducting coil, mounted perpendicular to our sample holder, and a flux line. We realize the parameter configuration shown in Supplementary Figure 3(c), with the transmon and the SQUID array in resonance, from which we extract the transmon SQUID array resonator coupling strength $2g_{\mathrm{tr,Sq}}/2\pi \sim 451.3 \pm 0.3$ MHz.

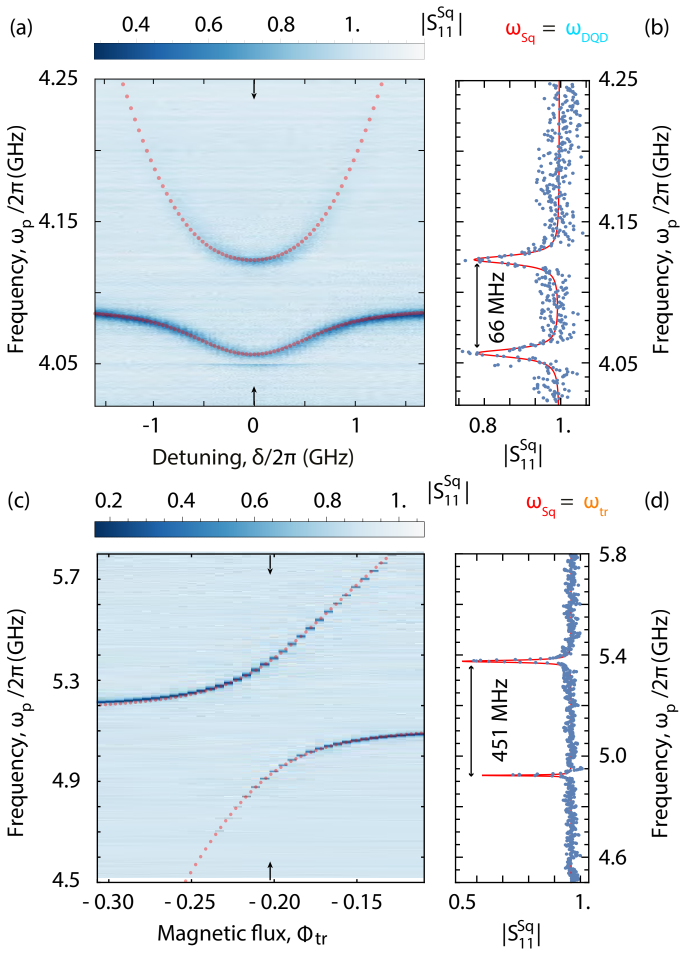

Supplementary Figure 3. Spectroscopy of the DQD-SQUID array and transmon-SQUID array vacuum Rabi mode splittings. (a) Reflection response $|S_{11}^{\rm Sq}|$ versus detuning δ of the DQD qubit hybridized with the resonator, resulting in a vacuum Rabi splitting at $\delta = 0$. The red points are the results of the systems spectrum simulation, according to the Eq. (1) with parameters $2t_{\rm c}/h = 4.090$ GHz, $\omega_{\rm r,Sq}/2\pi = 4.089$ GHz, $g_{\rm DQD,Sq}/2\pi = 33$ MHz and far detunned transmon at $\omega_{\rm tr}/2\pi \sim 1.720$ GHz. (b) $|S_{11}^{\rm Sq}|$ at $\delta = 0$ showing the vacuum Rabi splitting with $2g_{\rm DQD}/2\pi \sim 66.2 \pm 0.4$ MHz and an effective linewidth of $\delta\omega_{\rm DQD}/2\pi \sim 7.1 \pm 1.1$ MHz. The solid line is a fit to a sum of two Lorentzians. (c) Reflection response $|S_{11}^{\rm Sq}|$ versus the applied magnetic flux ($\Phi_{\rm tr}$) through the SQUID loop of the transmon qubit hybridized with the SQUID array resonator, resulting in an avoided crossing around $\omega_{\rm tr}/2\pi \sim \omega_{\rm r,Sq}/2\pi \sim 5.125$ GHz. Simulation parameters: $\omega_{\rm r,Sq}/2\pi = 5.181$ GHz, $g^0_{\rm tr,Sq}/2\pi = 271$ MHz [the couplings $g_{\rm tr,50\Omega}$ and $g_{\rm tr,Sq}$ vary with $\Phi_{\rm tr}$]. $\omega_{\rm DQD}/2\pi \sim 10.8$ GHz ($2t_{\rm c}/h \sim 4$ GHz and $\delta/h \sim 10$ GHz). (d) $|S_{11}^{\rm Sq}|$ at the flux indicated by the black arrows in panel (c) showing the vacuum Rabi splitting with $2g_{\rm tr}/2\pi \sim 451.3 \pm 0.3$ MHz and an effective linewidth of $\delta\omega_{\rm tr}/2\pi \sim 6.7 \pm 1.0$ MHz.

SUPPLEMENTARY NOTE 6

Simulation of the population transfer in Fig. 3(e)

The Markovian master equation used to fit the time-resolved transmon population oscillations in Fig. 3(e), treats both qubits as two-level systems in the dispersive regime with an exchange interaction $2J/2\pi = 21.8 \pm 0.1$ MHz consistent with the spectroscopically measured energy splitting $2J/2\pi \sim 21.1 \pm 0.2$ MHz [Fig. 3(c)]. It includes spontaneous emission and dephasing terms for the transmon and a dephasing term for the DQD. We determine the dephasing rates in independent measurements by measuring the DQD charge qubit linewidth $\gamma_2/2\pi = 2.7 \pm 0.4$ MHz and by performing Ramsey measurements of the transmon qubit resulting in $T_2^* = 127.6 \pm 8.8$ ns. The relaxation rate $T_1 = 188 \pm 2$ ns of the transmon is used as a fitting parameter in the simulation.

This relaxation rate is close to what expected by considering the Purcell decay ($\Gamma_{1,\rm tr} \approx k_{\rm tot} g^2_{\rm tr,Sq}/\Delta^2_{\rm tr,Sq} \sim 1$ MHz) of the transmon through the SQUID array resonator at the chosen detuning. Additionally, we include a finite time difference $\tau_0 = 30$ ns

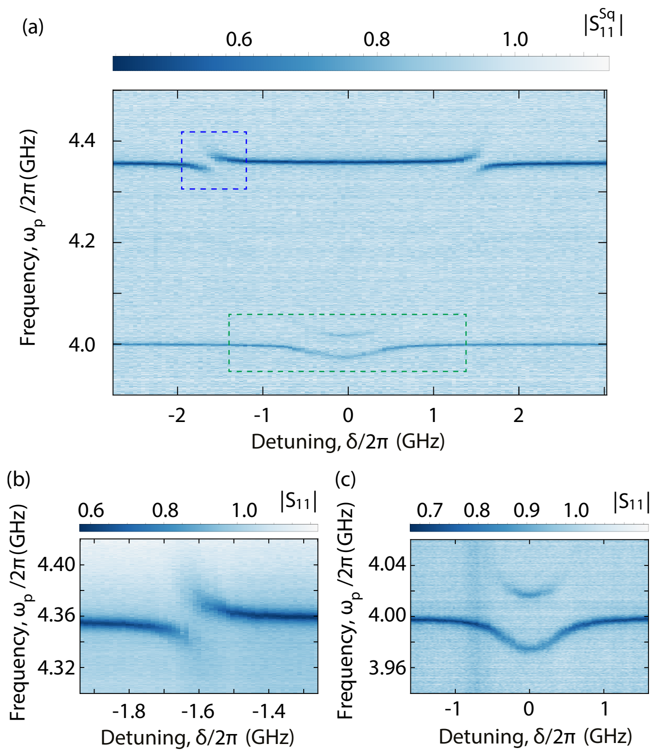

Supplementary Figure 4. (a) Reflectance $|S_{11}|$ of the SQUID array resonator hybridized with the transmon and DQD as a function of the DQD detuning δ, already reported in Fig. 2(b). (b) [(c)] Independent measurement of the region enclosed by the blue (green) dashed line in panel (a).

between the preparation pulse and the flux pulse as a fitting parameter. The flux pulse is simulated as a square pulse subject to a Gaussian filter with standard deviation $\sigma = 3\,\text{ns}$. We also take into account the finite rise time of the flux pulse, which in the experimental results most likely is due to the finite bandwidth (300 MHz) of the employed arbitrary wave form generator (Tektronix 5014 AWG).

SUPPLEMENTARY REFERENCES

[1] Koch, J. *et al.* Charge-insensitive qubit design derived from the Cooper pair box. *Phys. Rev. A* **76**, 042319 (2007).
[2] Stockklauser, A. *et al.* Strong coupling cavity QED with gate-defined double quantum dots enabled by a high impedance resonator. *Phys. Rev. X* **7**, 011030 (2017).
[3] Scarlino, P. *et al.* All-microwave control and dispersive readout of gate-defined quantum dot qubits in circuit quantum electrodynamics. *arXiv:1711.01906* (2017).
[4] Frey, T. *et al.* Dipole coupling of a double quantum dot to a microwave resonator. *Phys. Rev. Lett.* **108**, 046807 (2012).
